# Anæsthetic

**Published:** 1861-07

**Authors:** 


ANÆSTHETICS.—It is unfortunately too well known that with the many advantages belonging to the administration of *chloroform* as an anæsthetic is linked the possibility of an occasional fatal result, even in the most careful and experienced hands. There are very many who maintain that this drawback does not attach to *ether*. They assert its entire immunity from danger, although there may be some persons to whom the odor of it is offensive and irritating. It is less portable and agreeable to inhale than chloroform, but not more exciting or less efficient.

Dr. George Hayward (the first surgeon who performed a capital operation upon a patient rendered insensible by the inhalation of sulphuric ether), during a recent visit to Europe, instituted inquiries upon the point in question. Before his return across the Atlantic, he published some "Remarks on Anæsthesia and the Agents employed to produce it," in which he says: "I have not been able to find any well-attested case of death from its inhalation. There may have been such, but they have never come to my knowledge, though I have taken unwearied pains to obtain information on this point." In Mr. Ericksen's work it is affirmed of ether, that "no death has yet resulted from its use." The surgeons of Lyons declare that "since the adoption of ether in place of chloroform, the necrology of anæsthesia has not received an additional instance" in their city. Signor Palaschiano, of Naples, considers ether as infinitely safer than chloroform, in spite of the dangerous apparatus with which he administers it. Between these extreme schools of the belief in the perfect immunity of ether on the one hand, and its equal danger with chloroform without the latter's advantages on the other, has arisen a third party, which admits that ether is certainly not so hazardous as the other agent, but that fatal cases have occurred from its employment. They believe, further, that were ether more extensively used than it is, we should hear of a greater number of fatal cases from its inhalation. Ether is not employed as an anæsthetic agent to any extent in Great Britain and many parts of Continental Europe, but it is habitually used at Lyons and Naples, and is the only anæsthetic administered in the principal hospitals of the United States of America. We are informed that in the Massachusetts General Hospital at Boston, where it was first given, and has since then always been resorted to, no deaths have ever taken place, nor anxious apprehensions been excited, either during or after its inhalation.

An important question is, then, apparently undecided; and no means should be left untouched by which we may arrive at a definite opinion upon such experience as the profession has hitherto gained. The Boston Society for Medical Improvement has, we are glad to find, seriously taken up the matter, and has appointed a committee "to investigate the alleged deaths from the inhalation of sulphuric ether, and to report thereon." The vast interest attached to a truthful solution of the point at issue warrants us in urging our medical brethren to communicate to the chairman of the committee (Dr. Hodges) accounts of all cases which have already or shall hereafter come to their knowledge, in which a fatal issue appeared to result from the use of ether. It will be essential not only that the place, time, and circumstance of its occurrence be noted along with the mode of inhalation adopted, but that specific attention be paid to the following points:

1. The kind of ether used—whether *pure sulphuric ether, chloric ether*, or *ether combined with chloroform.*

2. The *period after inhalation* at which death occurred.—*London Lancet.*

